# *Polygala tenuifolia* extract inhibits lipid accumulation in 3T3-L1 adipocytes and high-fat diet–induced obese mouse model and affects hepatic transcriptome and gut microbiota profiles

**DOI:** 10.1080/16546628.2017.1379861

**Published:** 2017-10-05

**Authors:** Chun-Chung Wang, Jui-Hung Yen, Yi-Cheng Cheng, Chia-Yu Lin, Cheng-Ta Hsieh, Rung-Jiun Gau, Shu-Jiau Chiou, Hwan-You Chang

**Affiliations:** ^a^ Institute of Molecular Medicine, National Tsing Hua University, Hsinchu, Taiwan; ^b^ Biomedical Technology and Device Research Laboratory, Industrial Technology Research Institute, Hsinchu, Taiwan

**Keywords:** Obesity, lipid accumulation, liver transcriptome, *Polygala tenuifolia*, gut microbiota

## Abstract

Obesity, the excessive accumulation of lipids in the body, is closely associated with many prevalent human disorders. Continued efforts to identify plant extracts that exhibit anti-obesity effects have drawn much attention. This study investigated whether a *Polygala tenuifolia* extract (PTE) possesses anti-obesity activity and how PTE may affect liver gene expression and gut microbiota. We used 3T3-L1 adipocytes and a high-fat diet–induced obese mouse model to determine the effects of PTE on lipid accumulation. Next-generation sequencing analysis of liver gene expression and gut microbiota profiles following PTE treatment were conducted to elucidate possible mechanisms. We found that treatment of fully differentiated 3T3-L1 adipocytes with PTE inhibited lipid accumulation in the cells through reducing lipid formation and triglyceride content and by increasing lipase activity. No cytotoxicity was observed from the PTE treatment. After 5 weeks of treatment with PTE, the increased body weight, elevated serum triglyceride content, and liver steatosis in the high-fat diet–induced obese mice were each reduced. Liver transcriptomic analysis revealed that expression of genes involved in lipid and cholesterol metabolism was significantly altered. The low-grade chronic inflammation of obesity caused by a high-fat diet was also decreased after PTE treatment. In addition, treatment with PTE improved the relatively low Bacteroidetes/Firmicutes ratio in the gut of high-fat diet–fed mice through enrichment of the Proteobacteria population and reduction of the Deferribacteres population. In conclusion, treatment with PTE inhibited lipid accumulation by inducing the expression of the master transcription factor PPARα, attenuated the low-grade chronic inflammation of obesity, and also altered gut microbiota profiles. These results indicate that PTE has the potential to be developed into an anti-obesity food supplement and therapy.

**Abbreviations:** Abcg5: ATP-binding cassette subfamily G member 5; ALT: alanine aminotransferase; AMPK: adenosine monophosphate-activated protein kinase; AST: aspartate aminotransferase; B/F: Bacteroidetes to Firmicutes [ratio]; C/EBPα: CCAAT/enhancer-binding protein alpha; CR: creatinine; Cyp51: cytochrome P450 family 51; DMEM: Dulbecco’s modified Eagle’s medium; Fabp5: fatty acid-binding protein 5; FBS: fetal bovine serum; Fdps: farnesyl diphosphate synthase; Glc: Glucose; HFD: high-fat diet; GO: gene ontology; HPRT: hypoxanthine guanine phosphoribosyl transferase; IBMS: 3-isobutyl-1-methylxanthine; Idi1: isopentenyl-diphosphate delta isomerase 1; IL-1β: interleukin-1-beta; Lpin1: phosphatidic acid phosphohydrolase; LPS: lipopolysaccharide; Mvd: mevalonate diphosphate decarboxylase; ND: normal diet; OTU: operational taxonomic units; Pcsk9: proprotein convertase subtilisin/kexin 9; Pctp: phosphatidylcholine transfer protein; PPARα: peroxisome proliferator-activated receptor alpha; PPARγ: peroxisome proliferator-activated receptor gamma; PTE: *Polygala tenuifolia* extract; Saa1: serum amyloid A1; SD: standard deviation; SEM: standard error of the mean; Serpina12: serpin family member 12; Sqle: squalene monooxygenase; SREBP1C: sterol regulatory element-binding protein 1C; TCHO: total cholesterol; TG: triglyceride

## Introduction

Obesity is defined by the World Health Organization as a body mass index >30 kg/m^2^ [1]. In 2014, around 600 million people worldwide were estimated to be obese [2]. Obesity is associated with many common diseases, such as nonalcoholic fatty liver, hypertension, osteoarthritis, cardiovascular diseases, stroke, type 2 diabetes, and various cancers [3–6]. Due to the medical importance of obesity, considerable research has been devoted to developing appropriate treatments. Although several drugs have been approved by US Food and Drug Administration to treat obesity, their efficacy is often low and side effects are common [7,8]. Therefore, there is a great need for a well-tolerated treatment for obesity that has minimal side effects.

Recently, extracts from several plants have been reported to have anti-obesity effects. These plants include *Adenophora triphylla* [9], *Cudrania tricuspidata* [10], *Crinum asiaticum* [11], *Hibiscus sabdariffa* [12], green coffee bean [13], and *Tripterygium wilfordii* [14]. Several active anti-obesity ingredients present in plants have also been identified, such as saponins from *Glycyrrhiza uralensis* [15] and *Astragalus propinquus* [16], polysaccharides from *Lycium barbarum* [17,18] and *Schisandra chinensis* [18], and polyphenols from *Crataegus pinnatifida* Bunge [19]. These plant extracts and natural compounds have considerable potential to be further developed into effective therapies for obesity [20].

*Polygala tenuifolia*, also called Yuanzhi, is a traditional Chinese herbal medicine widely used in Asian countries to treat inflammation [21], depression [22], amnesia, neurasthenia, and insomnia, as well as for the prevention of dementia and memory loss [23,24]. Two herbal mixtures containing *P. tenuifolia*, SH21B and KBH-1, have been used to treat obesity in Korea [25]. SH21B was demonstrated to reduce fat accumulation in mice by inhibiting the expression of transcription factors involved in adipogenesis, such as peroxisome proliferator-activated receptor gamma (PPARγ), CCAAT/enhancer-binding protein alpha (C/EBPα), and sterol regulatory element-binding protein 1C (SREBP1C) [25]. The other herbal treatment, KBH-1, reduced lipid accumulation in rats by upregulating the adenosine monophosphate-activated protein kinase (AMPK) pathway, which resulted in the inhibition of fatty acid synthesis and adipogenic transcription factor PPAR [26,27]. However, the mechanism through which *P. tenuifolia* extract (PTE) alone inhibits fat accumulation has not been determined.

In the last decade, gut microbiota has been shown to be an important factor in metabolic dysregulation, including obesity and diabetes [28,29]. Among the four predominant bacterial phyla present in humans and mice [30] – namely Bacteroidetes, Firmicutes, Actinobacteria, and Proteobacteria – significant differences in the ratio of Bacteroidetes to Firmicutes (B/F) between obese and lean humans and rodents have been noted [31,32]. A relatively lower population of Bacteroidetes with a corresponding increase in Firmicutes was noted in some studies when comparing obese and lean individuals [33,34]. However, shifts to lower B/F ratios have also been reported elsewhere, suggesting that the functional role of gut microbiota in obesity remains unclear and must be investigated [35,36]. The microbial components that have been proposed to affect host metabolism include short-chain fatty acids and lipopolysaccharides (LPS) [37]. The high-fat diet (HFD)–induced gut microbiota distribution changes in mice may increase LPS production [38]. In addition, one recent study demonstrated that mice receiving low doses of LPS developed an obese phenotype similar to those fed a HFD [39]. The association between LPS and obesity is less clear, although the low-grade inflammatory effects triggered by LPS are probably involved.

This study investigated whether PTE possesses anti-obesity activity and how this activity affects liver gene expression and gut microbiota. We demonstrated that PTE exhibited strong activity, reducing lipid accumulation both *in vitro* and *in vivo*. Our experiments also yielded evidence that the anti-obesity effect of PTE treatment is probably caused by the modulation of hepatic gene expression and gut microbiota composition in mice.

## Materials and methods

### Materials and reagents

The roots of PTE were obtained from local Chinese medicine suppliers. Preadipocyte 3T3-L1 cells (BCRC 60159) were obtained from the Bioresource Collection and Research Center (Food Industry Research and Development Institute, Hsinchu, Taiwan). Dexamethasone, 3-isobutyl-1-methylxanthine (IBMS), and insulin were purchased from Sigma-Aldrich (St Louis, MO, USA). Fetal bovine serum (FBS) and Dulbecco’s modified Eagle’s medium (DMEM) were purchased from Gibco (Waltham, MA, USA).

### Preparation of PTE

Raw PTE was dried and ground into powder (20–40 mesh) using a commercial grinder. Next, 100 g of the PTE powder was mixed with 1 L of distilled water and stirred extensively at room temperature. The mixture was subjected to heat reflux extraction at 100°C for 1 h. The extraction efficiency (weight of the extract after extraction/weight of the raw materials before extraction) was ~15%. The extract was further concentrated to a final volume of 50 mL using a rotary evaporator at 70°C. Lyophilization of the concentrated extract was performed using the following program: −20 to −10°C for 2 h; −10 to −5°C for 2.5 h; −5 to 4°C for 3 h; 4 to 12°C for 5 h; 12 to 18°C for 5 h; 18 to 22°C for 5 h; and 22 to 28°C for 5 h.

### Culture and differentiation of 3T3-L1 cells into adipocytes

3T3-L1 preadipocyte cells were cultured in DMEM containing 10% FBS at 37°C in 5% CO_2_. Cells were maintained at 90% confluency in T75 flasks and subcultured twice per week. Prior to induction of differentiation, 3T3-L1 cells were seeded at a density of 2 × 10^4^ cells/well into a 12-well culture plate. The cells were then cultured in differentiation medium containing DMEM supplemented with 10% FBS, 0.5 mM IBMS, 1 μM dexamethasone, 10 μg/mL insulin, and 2 μM rosiglitazone for 48 h [40]. The medium was then replaced with DMEM containing 10% FBS and 10 μg/mL insulin for 4 days. The medium was changed every 2 days until the cells were analyzed.

### Cell viability

Fully differentiated 3T3-L1 cells were seeded at a density of 2 × 10^4^ cells/well in a 96-well culture plate. Viable cells after treatment with different concentrations of PTE for 48 h were evaluated using MTT colorimetric assay.

### Oil Red O staining

Oil Red O staining was performed on fully differentiated 3T3-L1 cells in the presence or absence of PTE for 4 days. The cells were washed twice with PBS, fixed with 3.7% formaldehyde at room temperature for 20 min, and stained with Oil Red O (Sigma-Aldrich) at 50°C for 10 min. Cells were then washed with 60% isopropanol three times, and 98% isopropanol was added to dissolve the Oil Red O dye. The amount of Oil Red O dye was quantitated by absorbance at 492 nm.

### Measurement of lipolysis and triglyceride content

Lipolysis activity of 3T3-L1 cells was determined by measuring the amount of glycerol released into the incubation medium (GY105; Randox, England). The cells were then disrupted by adding 1% Triton X-100 in PBS and the triglyceride content was measured using a TR212 kit (Randox).

### HFD-induced obese mouse model

All animal experimental procedures followed the protocols approved by the Institutional Animal Care and Use Committee of the Industrial Technology Research Institute (ITRI-IACUC-2014-73V2) in Hsinchu, Taiwan. Four-week-old male ICR mice were purchased from BioLASCO (Taiwan) and housed in standard laboratory conditions (21–25°C, 40–70% humidity, and 12-h light/dark cycle). All mice were randomly divided into two groups as follows: normal diet (ND; 10 kcal % fat diet, standard laboratory diet #1320, Altromin, Germany) and HFD (60 kcal % fat diet, Research Diets D12492, Open Source, USA). After 4 weeks on the HFD, obese mice with a weight 10% higher than that of the ND group mice were selected and divided into two groups: HFD control group, which was given distilled water orally once daily for 5 weeks (n = 10), and PTE treatment group, which was given PTE dissolved in distilled water orally once daily for 5 weeks (n = 10).

### Measurement of body weight, serum parameters, and epididymal fat content

The body weight and feed intake of the mice were monitored three times every week. Changes to body weight were calculated as the percentage increase compared with weight before treatment (on Day 0). After 5 weeks of treatment, all mice were fasted for 6 h and then sacrificed through CO_2_ overdose. Heart blood was collected and serum was obtained by centrifugation at 6000 rpm at room temperature for 15 min. Alanine aminotransferase (ALT), aspartate aminotransferase (AST), creatinine (CR), triglyceride (TG), total cholesterol (TCHO), and glucose (Glc) were analyzed using a FUJI DRI-CHEM 4000i biochemistry analyzer. Epididymal fat was collected and weighed, and the corresponding data are represented as percentage of body weight. The liver was collected and divided into two parts: one was fixed in the 4% formalin and the other was frozen in liquid nitrogen and stored at −80°C for RNA extraction.

### Histological examination

Mouse liver tissues were collected, fixed in 4% formalin for 24 h, and then embedded in paraffin. Tissue sections 5 μm in thickness were obtained using a microtome and stained with hematoxylin and eosin. Images of the tissue sections were taken using a light microscope at different magnifications. The severity of liver lesions was determined by a pathologist according to the standard method [41] using the following scores: 1 = minimal (<1%); 2 = slight (1–25%); 3 = moderate (26–50%); 4 = moderate/severe (51–75%); 5 = severe/high (76–100%).

### RNA extraction and real-time quantitative PCR analysis

Total RNA was extracted from 100 mg of mouse liver tissue using an RNeasy kit (Qiagen, Germany), and 2 μg of RNA was reverse transcribed using a QuantiTect Reverse Transcription Kit (Qiagen). Real-time reverse transcription PCR was performed using the CFX Connect Real-Time PCR System (Bio-Rad, Hercules, CA, USA). The final volume of the PCR reaction mixture was 10 μL and comprised SYBR green master mix (iQ™ SYBR® Green Supermix, Bio-Rad), primer sets for each candidate gene, distilled water, and cDNA. The reaction mixtures were preheated at 95°C for 3 min and then subjected to 40 cycles of melting at 95°C for 10 s, annealing/extension at 55°C for 10 s, and elongation at 72°C for 30 s. All reactions were performed in triplicate and normalized to β-actin and hypoxanthine guanine phosphoribosyl transferase (HPRT) genes. The data were analyzed using Bio-Rad CFX Manager 3.1 software (Bio-Rad) and are presented as fold changes of the normalized mRNA amounts of the PTE treatment group to those of the HFD control group.

### Differential gene expression analysis (RNA-seq quantification)

Differential gene expression analysis was performed through RNA-seq quantification [42] following the protocol of Illumina performed by Genomics (Taiwan) to obtain ~10 million reads for each sample. Data analysis first filtered low-quality reads, which was followed by alignment of the sequence of each read against the mouse genome using reference annotation. The gene expression profiles in the PTE treatment and HFD control groups were analyzed and compared. The genes showing differential expression were further categorized through gene ontology (GO) analysis.

### Mouse gut microbiota analysis

Mouse gut microbiota profiles were determined through 16S rRNA gene sequencing of genomic DNA from stool [43]. After 5-week PTE treatment, DNA was extracted from mouse stool samples using a QIAamp DNA Stool Mini Kit (Qiagen, Germany). The Illumina V3 forward 5ʹ-CCTACGGGNGGCWGCAG-3ʹ and V4 reverse 5ʹ-GACTACHVGGGTATCTAATCC-3ʹ PCR primers were used for amplifying bacterial 16S rRNA variable regions. The amplicons were then subjected to sequencing (Genomics, Taiwan) and the data analyzed. Briefly, the following steps were performed to obtain the final effective reads: each sample was demultiplexed by dual-index using an in-house written script; paired-end reads were joined using the PEAR program (Exelixis Lab, Heidelberg Institute for Theoretical Studies, German); primer sequences were trimmed from joined reads using the AlienTrimmer program; low-quality end sequences were trimmed off using sliding windows (five nucleotides) with an average quality value <10, and short sequences of <200 nucleotides were screened out using the Trimmomatic program; and chimeric reads were filtered out using the Mothur v.1.33.3 software package. All effective reads were calculated for pairwise distances between aligned DNA sequences with a cutoff of 0.03, then clustered into operational taxonomic units (OTU) using the average neighbor algorithm with a cutoff of 0.03, and finally the OTUs were classified for taxonomic assignment. The bioinformatics software packages Mothur v.1.33.3 and QIIME v1.80 were then used for taxonomic composition of the 16S rRNA sequences using the Greengenes 16S rRNA Taxonomy Database (gg_13_8).

### Statistical analysis

For *in vitro* assays, data are represented as mean ± standard deviation (SD). For *in vivo* assays, data are represented as mean ± standard error of the mean (SEM). Statistical analysis was performed using Student’s *t*-test. Significant differences between groups were determined using one-way ANOVA and Dunnett’s multiple comparisons test was used to perform *post hoc* analysis. In the gut microbiota distribution analysis, the percent distribution of the gut microbiota, richness index (number of observed species), and alpha diversity metrics (Shannon index) of each group were calculated and analyzed using the Pearson’s chi-squared test. Differences between the ND group and HFD control group (denoted ^#^) and between the PTE group and HFD control group (denoted *) were considered significant at p < 0.05 (^#^/*), p < 0.01 (^##^/**), and p < 0.001 (^###^/***).

## Results

### Treatment with PTE reduced lipid accumulation in differentiated 3T3- L1 adipocytes

**Figure 1.**
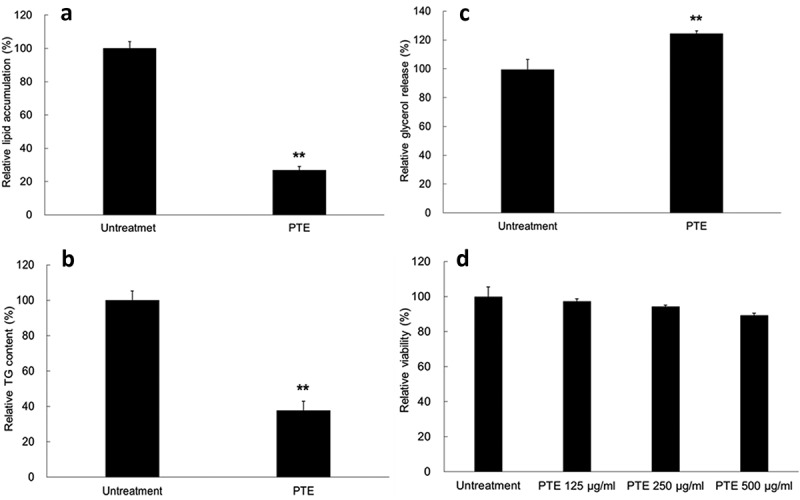
Treatment with PTE inhibited lipid accumulation in 3T3-L1 adipocytes. Undifferentiated 3T3-L1 cells and their differentiated derivatives were used to determine the effect of 500 μg/mL PTE treatment on lipid accumulation (a), intracellular triglyceride content (b), lipase activity (c), and cytotoxicity (d). Percentages were calculated as relative to the adipocyte group without PTE treatment (Untreatment). Data are presented as mean ± SD. Significant differences from the control group are indicated by * p < 0.05, ** p < 0.01, and *** p < 0.001.

Differentiated 3T3-L1 adipocytes are a useful model for screening compounds modulating lipid accumulation. As shown in Figure 1(a), lipid accumulation in the adipocytes was 26.9% of that in the control group after 500 μg/mL PTE treatment was administered for 4 days. Similarly, the TG content in the PTE-treated differentiated adipocytes was 37.8% that of the control group (Figure 1(b)). The lipolysis activity of the cells, determined by glycerol release, was 24.4% higher in the PTE treatment group relative to the HFD control group (Figure 1(c)). In addition, no obvious cytotoxicity was detected even after treatment with a relatively high concentration of PTE (500 μg/mL) (Figure 1(d)). These results indicate that treatment of 3T3-L1 cells with PTE substantially inhibited lipid accumulation in cells without causing cytotoxicity.

### PTE inhibited body weight increases and lipid accumulation in HFD-induced obese mice

**Figure 2.**
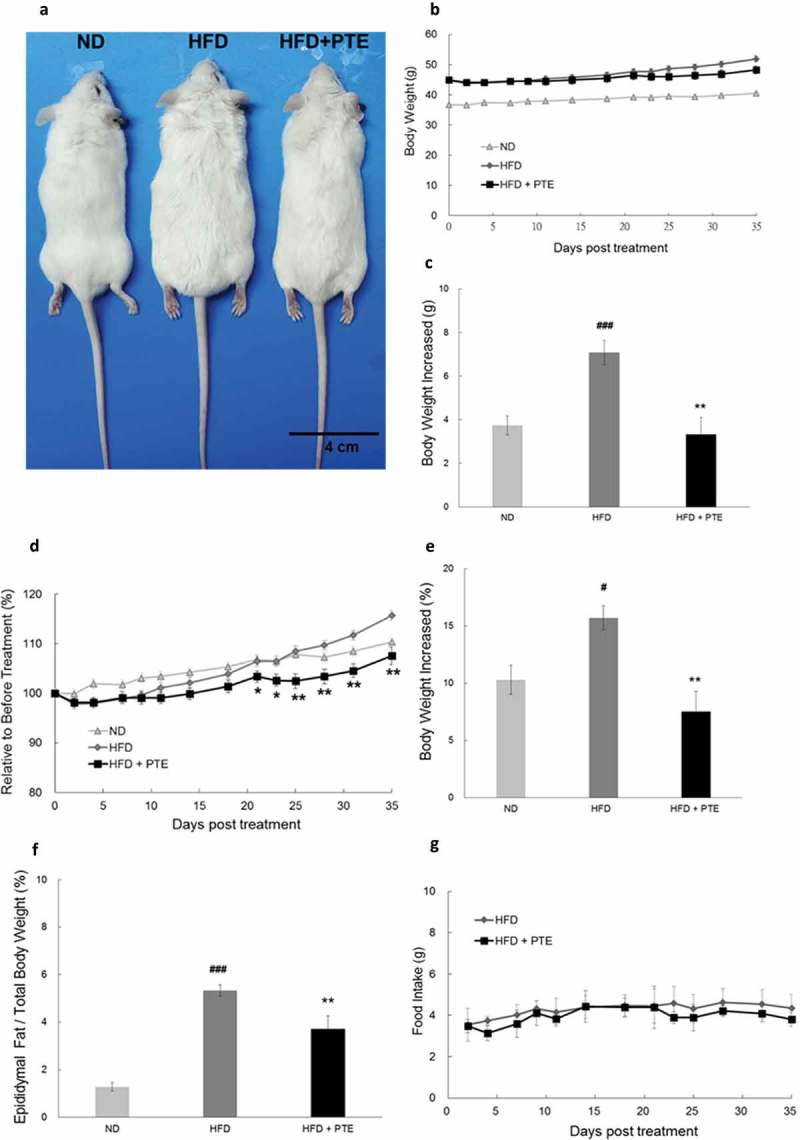
Treatment with PTE inhibited lipid accumulation in HFD-induced obese mice without altering food intake. Obese mice derived from HFD feeding for 4 weeks were divided into the HFD control group (n = 10) and PTE treatment group (n = 10). Mice in the HFD control group received distilled water, whereas mice in the PTE group were administered 250 mg/kg PTE orally daily for 5 weeks. The normal diet group (ND, n = 8) were fed a ND throughout the entire experiment period. (a) Phenotypes of mice in ND, HFD control, and PTE treatment groups. Scale bar = 4 cm. (b) Body weight (g) plot of each experimental group during the treatment period. (c) Average increased body weight (g) of each experimental group. (d) Body weight (%) plot at each measurement day relative to the first day of treatment during the experimental period. (e) Mean relative increased body weight (%) of each group after treatment for 5 weeks. (f) Weight of epididymal fat of mice in each experimental group after treatment for 5 weeks. (g) Amounts of food intake in the HFD control and PTE treatment groups. Data are presented as mean ± SEM. Significant differences between the ND group and HFD control group are indicated by ^#^p < 0.05, ^##^p < 0.01, and ^###^p < 0.001. Significant differences between the PTE group and HFD control group are indicated by *p < 0.05, **p < 0.01, and ***p < 0.001.

To investigate the effects of PTE *in vivo*, we fed mice a HFD for 4 weeks to induce obesity. Obese mice, which had a body weight that was 10% higher than the mice fed a ND, were selected and divided into two groups: HFD control, and PTE treatment. Both groups were on the HFD, but mice in the PTE treatment group were given 250 mg/kg PTE daily for 5 weeks. As demonstrated in Figure 2(a), the body size of the mice treated with PTE was considerably smaller than that of the HFD control mice. The average body weight of mice was 40.4 ± 0.7 g, 51.9 ± 1.3 g, and 48.3 ± 1.5 g in the ND, HFD control, and PTE treatment groups, respectively (Figure 2(b)). In the 5-week treatment period, the average body weight increases of the ND and HFD controls were 3.7 ± 0.4 g and 7.08 ± 0.6 g, respectively. By contrast, the weight of mice in the PTE group only increased by 3.3 ± 0.8 g, indicating the effectiveness of PTE in body weight control (p < 0.01) (Figure 2(c)). Figure 2(d) plots the average percentage body weight change in the PTE treatment group mice (103.4% ± 1.2%) after 21 days of treatment, which was significantly lower (p < 0.05) than that of the mice in the HFD control group (106.5% ± 0.9%). At the end of the 5-week treatment, body weight gain in the PTE group was 7.5% ± 1.7% (Figure 2(e)), significantly lower than that in the ND group (10.3% ± 1.3%) and HFD control group (15.7% ± 1.1%). Furthermore, the percentage of epididymal fat in the ND group and HFD control group mice was approximately 1.2 ± 0.2% and 5.3 ± 0.2%, respectively, whereas that in the PTE group was significantly lower at 3.7 ± 0.5% compared with the HFD control group (p < 0.01) (Figure 2(f)). These results demonstrated that oral administration of PTE (250 mg/kg) daily for 5 weeks significantly inhibited lipid accumulation in HFD-fed mice. No significant difference in feed intake was observed between mice in the HFD control and PTE treatment groups (Figure 2(g)), indicating that the anti-obesity effect of PTE was not a result of appetite reduction.

### PTE reduced triglyceride levels in the serum of HFD-induced obese mice and exhibited no hepatotoxicity or nephrotoxicity

**Figure 3.**
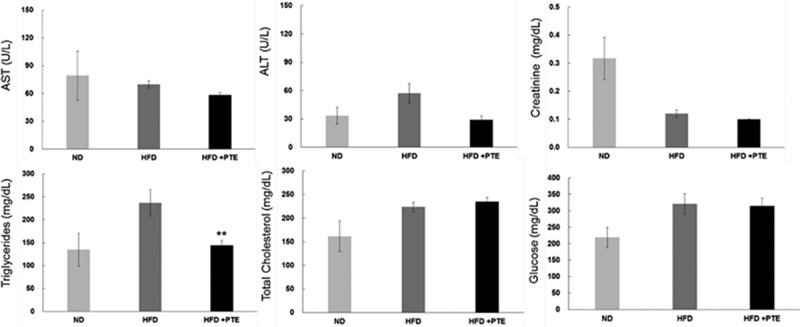
Treatment with PTE reduced serum triglyceride levels without producing hepatotoxicity or nephrotoxicity. Serum was collected from each experimental group and the following parameters were determined: alanine aminotransferase (ALT), aspartate aminotransferase (AST), nephrotoxicity indicator (creatinine), triglycerides, total cholesterol, and glucose. Data are presented as the mean ± SEM. Significant differences between the PTE treatment group and HFD control group are indicated by *p < 0.05, **p < 0.01, and ***p < 0.001.

In addition to cytotoxicity, this study further evaluated whether administering PTE would produce any adverse effects. Figure 3 illustrates the levels of several major liver and kidney markers in the serum of mice in the PTE treatment group at the end of experiment. The levels of these markers – which included ALT, AST, CR, TCHO, and Glc – in the PTE, HFD control, and ND groups were measured. The elevated TG level in the serum of mice in the HFD group (236.9 ± 28.6 mg/dL) decreased significantly (144.0 ± 11.0 mg/dL) after PTE treatment (p < 0.01). This finding is consistent with the hypothesis that PTE inhibited lipid accumulation.

### Treatment with PTE attenuated liver steatosis in HFD-induced obese mice

**Figure 4.**
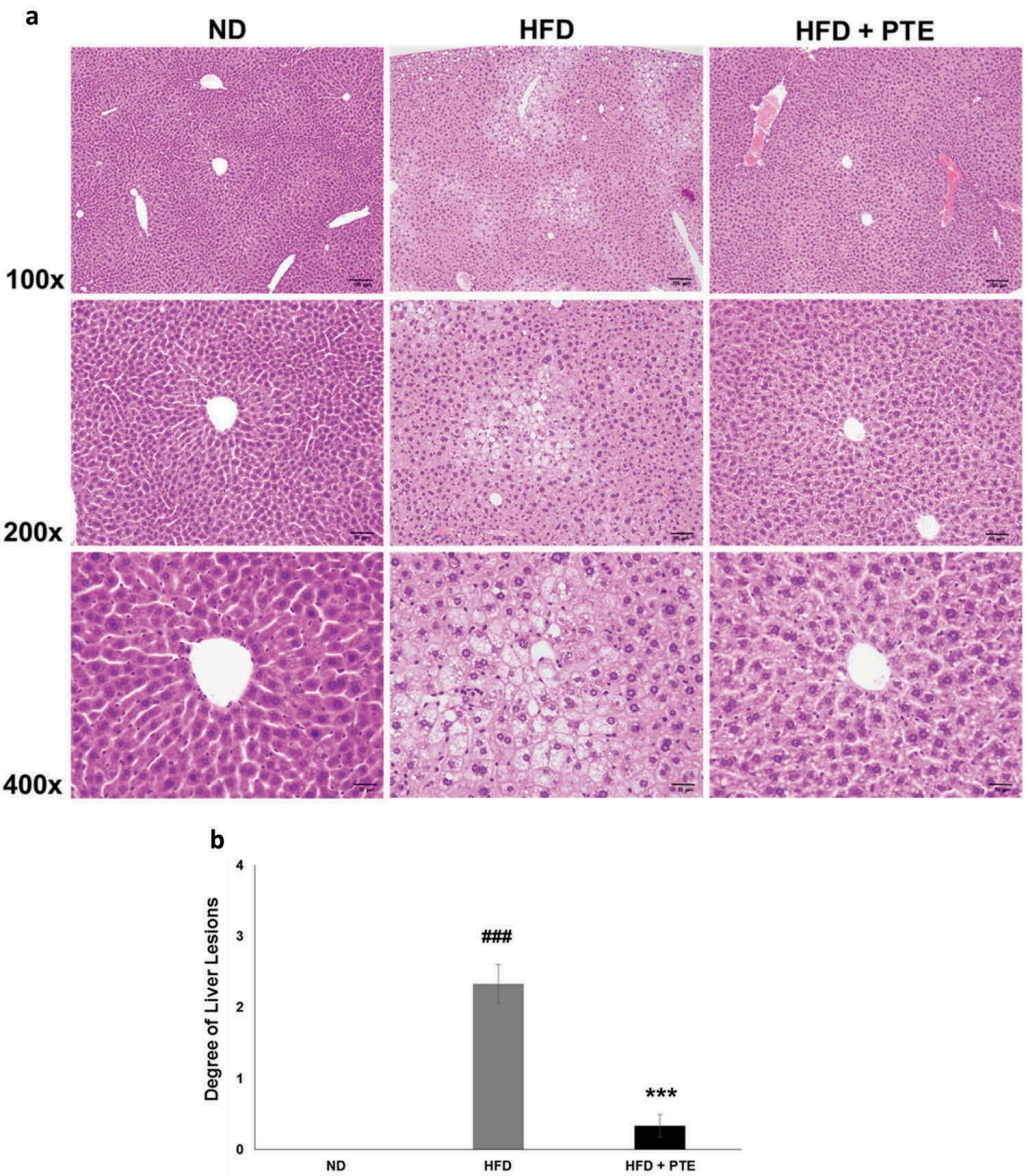
Treatment with PTE attenuated HFD-induced liver lesion. Comparison of histopathological alterations of the liver in the ND, HFD control, and PTE groups. (a) Tissue sections were prepared and stained with hematoxylin and eosin. The sections were photographed at 100×, 200×, and 400× magnifications. Scale bars = 100, 50, and 20 μm at each magnification, respectively. (b) Quantification of liver lesion histology. Significant differences between ND and HFD group are indicated by ^###^p < 0.001; HFD and PTE group are indicated by ***p < 0.001.

The histology of mouse liver tissues in each of the experimental groups was examined after the 5-week treatment (Figure 4(a)). As demonstrated in Figure 4(b), HFD feeding for 5 weeks resulted in multifocal and moderate microvesicular fatty changes in the liver of the HFD control group. The average liver lesion score of the HFD group was 2.3 ± 0.3, significantly higher than that of the ND group (p < 0.001). However, after 5 weeks of PTE treatment, the microvesicular fatty change observed was reduced and the liver lesion score had decreased to 0.3 ± 0.2, which was significantly (p < 0.001) lower than that of the HFD group. This histopathological finding strongly suggests that PTE has a protective effect on HFD-induced chronic liver injuries.

### Effect of PTE treatment on liver gene expression in HFD-induced obese mice

To investigate the effects of PTE treatment on liver gene expression, an RNA-seq study was performed using RNA samples pooled from three independent mouse livers from each experimental group. We excluded genes with a transcript read number less than 15 and selected those with fold changes ≥1.5-fold between the experimental groups. Additional files (Table S1A and S1B) compile the 55 upregulated and 72 downregulated genes in the PTE group, and the top 30 up- and downregulated genes are listed in Tables 1 and 2, respectively. These genes were further categorized using GO analysis with an emphasis on those related to lipid metabolism and catabolism, as shown in Table 3.

**Table 1.** Top 30 downregulated genes upon PTE treatment.

Gene	Fold change^a^
Rpl14-ps1	0.05
Marco	0.07
BC021614	0.12
Creld2	0.23
Mup19	0.24
2310057J18Rik	0.26
Myom3	0.26
Mvd	0.28
Il1β	0.30
Sqle	0.31
Pira1	0.31
Fam25c	0.31
Lcn2	0.32
Cyp2d12	0.35
Slc17a9	0.35
Gale	0.36
Igsf23	0.37
Serpina3g	0.38
Xlr3a	0.39
Rsc1a1	0.40
Igsf6	0.40
Acnat2	0.41
Syvn1	0.41
Rhbg	0.42
Eif3j2	0.42
Saa2	0.42
Lad1	0.42
Hyou1	0.42
Pqlc1	0.43
Manf	0.43

**^a^**Fold change represents the ratio of PTE group to HFD group.

**Table 2.** Top 30 upregulated genes upon PTE treatment.

Gene	Fold change^a^
Cd34	4.88
Pctp	4.77
Cela1	4.44
Cyp2a5	4.16
Hspa1a	3.57
Tff3	3.43
Rassf4	3.42
Lpin1	3.29
Fam171b	3.26
Afap1l1	3.14
Rgs16	2.99
Cyp2a22	2.92
Vsig10	2.85
Cyp39a1	2.83
Hspa1b	2.82
Cfap53	2.76
Grem2	2.76
Akr1e1	2.64
Ahcy	2.43
Lyve1	2.36
Alas1	2.33
PPARα	2.28
Cyp2a12	2.26
Plxna2	2.23
Rsph3b	2.20
Ceacam2	2.14
Smad9	2.13
Gm10052	2.12
Dsg1c	2.10
Slco1a4	2.10

**^a^**Fold change represents the ratio of PTE group to HFD group.

**Table 3.** Gene ontology (GO) analysis of the differential expression genes.

GO ID/Function	Genes
0006629/Lipid metabolic process	Pcsk9/Fdps/Elovl3/Cyp2a12/Cyp2a5/Cyp51/Lpin1/G6pc/Il1b/Fabp5/Pctp/Mvd/Saa1/Sqle/Acnat2/Cyp2a22/Hsd17b6/Slco1a4/Idi1/Cyp2d12/Cyp39a1/Msmo1/Serpina12/Gpcpd1
0044255/Cellular lipid metabolic process	Pcsk9/Fdps/Elovl3/Cyp2a12/Cyp2a5/Lpin1/G6pc/Fabp5/Mvd/Acnat2/Cyp2a22/Idi1/Cyp2d12/Msmo1/Serpina12/Gpcpd1
0008610/Lipid biosynthetic process	Fdps/Elovl3/Cyp51/Lpin1/Il1b/Fabp5/Mvd/Idi1/Cyp39a1/Msmo1/Serpina12
0016042/Lipid catabolic process	Lpin1/Il1b/Cyp39a1/Gpcpd1
0044241/Lipid digestion	Abcg5
0006631/Fatty acid metabolic process	Elovl3/Cyp2a12/Cyp2a5/Lpin1/Acnat2/Cyp2a22/Cyp2d12/Msmo1
0022600/Digestive system process	Abcg5/Rsc1a1
1,901,568/Fatty acid derivative metabolic process	Cyp2a12/Cyp2a5/Cyp2a22/Cyp2d12
0050892/Intestinal absorption	Abcg5/Rsc1a1
0033559/Unsaturated fatty acid metabolic process	Cyp2a12/Cyp2a5/Cyp2a22/Cyp2d12

Lipid metabolism–related genes with 1.5-fold up- or downregulation upon PTE treatment are listed.

A large proportion of the top PTE downregulated genes were related to lipid and cholesterol biosynthesis. PTE downregulated genes include serpin family member 12 (Serpina12), mevalonate diphosphate decarboxylase (Mvd), proprotein convertase subtilisin/kexin 9 (Pcsk9), squalene monooxygenase (Sqle), farnesyl diphosphate synthase (Fdps), isopentenyl-diphosphate delta isomerase 1 (Idi1), and cytochrome P450 family 51 (Cyp51) (Table 1). Similarly, PTE treatment activated the expression of several genes that facilitate lipid or cholesterol transfer and lipid oxidation, such as phosphatidylcholine transfer protein (Pctp), ATP-binding cassette subfamily G member 5 (Abcg5), cytochrome P450 family 2a (Cyp2a5, Cyp2a22, and Cyp2a12), phosphatidic acid phosphohydrolase (Lpin1), and fatty acid-binding protein 5 (Fabp5) (Table 2). Compellingly, the expression of two inflammation-related genes, interleukin-1-beta (IL-1β) and serum amyloid A1 (Saa1), was downregulated 0.29- and 0.47-fold (Table 1), respectively, by PTE treatment, suggesting that PTE exhibits anti-inflammatory activity.

To identify the key regulator responsible for PTE treatment, we investigated the gene expression of the major transcription factors (Table 4) that regulate lipid biosynthesis, metabolism, and oxidation. The most notable change in the expression level of transcription factors regulating lipolysis was that in PPARα. PPARα expression was upregulated 2.28-fold when the HFD-induced obese mice were administered PTE for 5 weeks. The expression of three transcription factors, Cebpα, Srebf1, and PPARγ, has been reported previously to be inhibited by PTE-containing herb mixtures SH21B and KBH-1 [25,27]. However, the expression levels of these transcription factors in mouse liver were not altered significantly after treatment with PTE.

**Table 4.** Expression levels of gene encoding transcription factors involved in adipogenesis.

Full name	Gene	Fold change^a^
Insulin-induced gene 1	Insig1	0.80
Nuclear receptor subfamily 1, group H, member 3	Nr1h3	0.92
Hypoxia inducible factor 1, alpha subunit	Hif1α	0.93
CCAAT/enhancer binding protein (C/EBP) alpha	Cebpα	1.03
Peroxisome proliferator activated receptor gamma	Pparγ	1.03
Peroxisome proliferator activated receptor alpha	Pparα	2.28
Catenin beta 1	Ctnnb1	1.05
Upstream transcription factor 1	Usf1	1.07
Retinoblastoma 1	Rb1	1.17
Endothelial PAS domain protein 1	Epas1	1.18
Nuclear receptor interacting protein 1	Nrip1	1.23
Forkhead box O1	Foxo1	1.27
Kruppel-like factor 15	Klf15	1.34
Sterol regulatory element binding protein 1	Srebf1	1.37
Kruppel-like factor 6	Klf6	1.42
High mobility group AT-hook 1	Hmga1	1.52

**^a^**Fold change indicates the ratio of transcription reads of a gene in the PTE group to that in the HFD control group.

To verify the differentially expressed genes identified by RNA-seq, real-time quantitative PCR analysis was performed and analyzed. As demonstrated in Figure 5(a), elevated expression of Abcg5 (1.89-fold), Lpin1 (1.51-fold), Pctp (4.32-fold), and PPARα (3.46-fold) were observed using real-time PCR. Most of the PTE downregulated genes – including IL-1β (0.47-fold), Idi1 (0.45-fold), Fdps (0.41-fold), Sqle (0.28-fold), Cyp51 (0.55-fold), Mvd (0.47-fold), and PPARγ (0.72-fold) – displayed a similar trend as determined using real-time PCR. No significant changes between the HFD control and PTE treatment groups were noted using either RNA-seq or real-time PCR analysis for the expression of several genes that have been related to lipid accumulation in previous studies [25,44], including acetyl-CoA carboxylase 1 (Acaca), ATP-citrate lyase (Acly), fatty acid synthase (Fasn), low-density lipoprotein receptor (Ldlr), peroxisomal acyl-coenzyme A oxidase 1 (Acox1), and forkhead box O1 (Foxo1). Figure 5(b) shows the genes whose expression was altered by PTE treatment according to both the real-time quantitative PCR and RNA-seq results. These differentially expressed genes are promising targets for development of future anti-obesity treatments.

**Figure 5.**
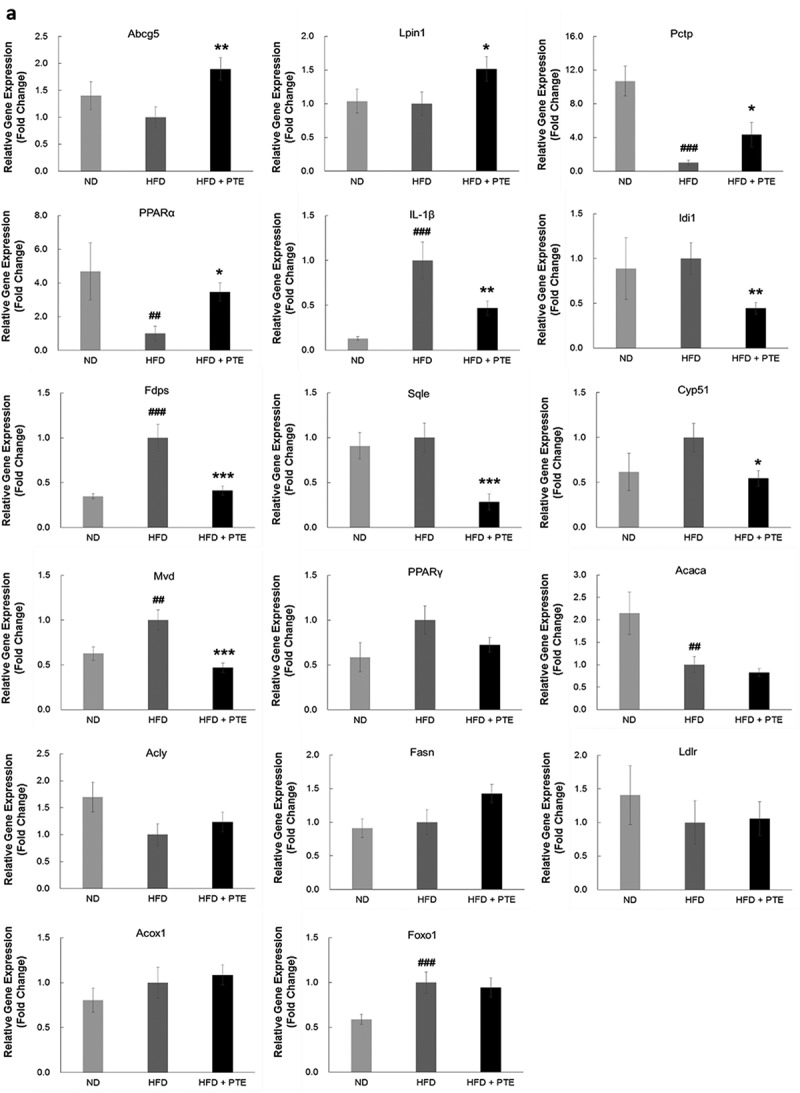
Confirmation of PTE-induced differentially expressed genes using real-time PCR. (a) The genes showing differential expression in the RNA-seq study were verified using real-time quantitative PCR. Expression of each gene was normalized to that of β-actin and hypoxanthine guanine phosphoribosyl transferase (HPRT) genes. (b) Dot plot displaying the relative degrees of differential expression of selected genes. Fold changes indicate the gene expression in the PTE treatment group relative to that in the HFD control group. Data are presented as the mean ± SEM. Significant differences between the ND group and HFD control group are indicated by ^#^p < 0.05, ^##^p < 0.01, and ^###^p < 0.001. Significant differences between the PTE group and HFD control group are indicated by *p < 0.05, **p < 0.01, and ***p < 0.001.

**Figure 5.**
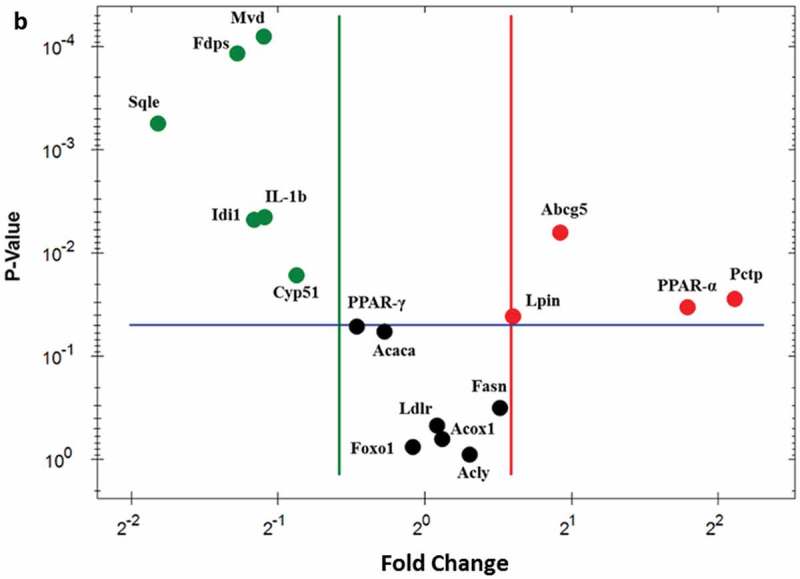
Continued.

### Effect of PTE on gut microbiota composition in HFD-induced obese mice

**Figure 6.**
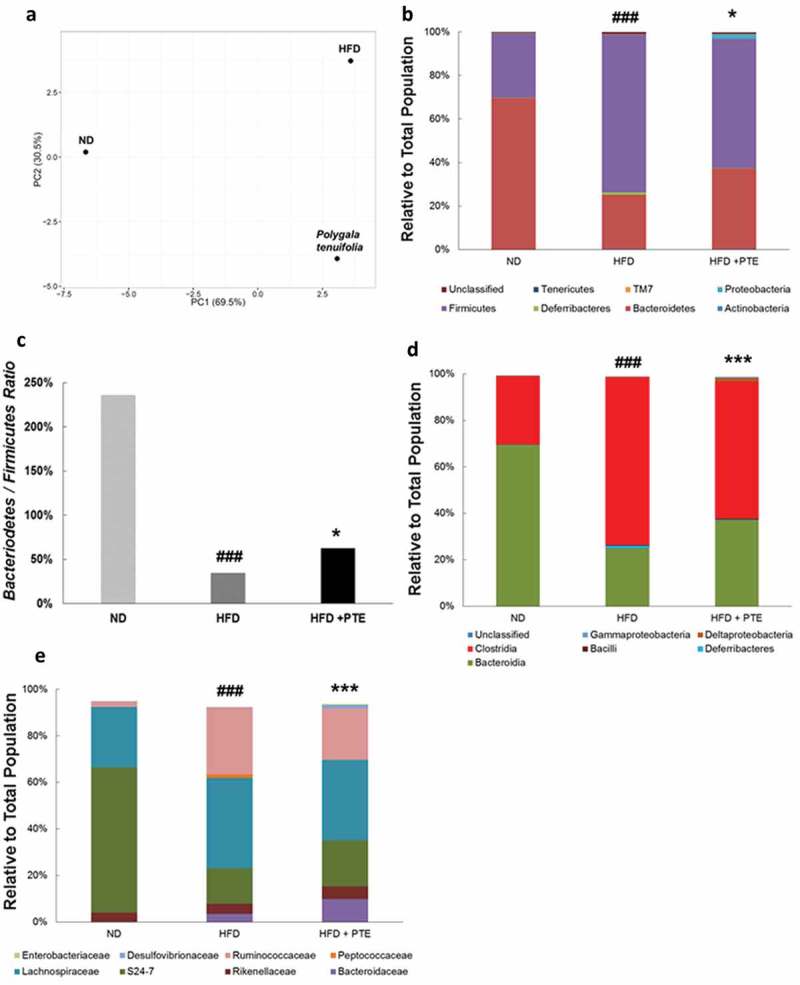
Gut microbiota profiles in HFD control and PTE-treated mice. (a) Diversity of gut microbiota in ND, HFD control, and PTE treatment groups was determined using principal component analysis. The dominant bacterial population in each group at the phylum level (b), class level (d), and family level (e) are shown. (c) Bacteroidetes to Firmicutes ratio of ND, HFD control, and PTE-treated mice. Significant differences between the ND group and HFD control are indicated by ^#^p < 0.05, ^##^p < 0.01, and ^###^p < 0.001. Significant differences between the PTE group and HFD control are indicated by *p < 0.05, **p < 0.01, and ***p < 0.001.

To investigate whether the gut microbiota profile was altered in the mice that received PTE treatment, we collected the stool of the ND group (n = 8), HFD control group (n = 10), and PTE treatment group (n = 10) after a 5-week treatment course. Genomic DNA was extracted from the stools, and the distribution profiles of different bacterial groups were determined based on 16S rRNA sequences. Figure 6(a) shows the results of principal component analysis, which demonstrate obvious differences in gut microbiota profile between the ND and HFD control groups. Notably, significant differences in gut microbiota profiles between the ND, HFD control, and PTE treatment groups were observed. The richness index (number of observed species) and alpha diversity metrics (Shannon index) of the gut microbiota in each group were also calculated (Figure S1). The findings indicate that the differences in gut microbiota between the ND, HFD control, and PTE treatment groups were highly significant (p < 0.001). Figure 6(b) displays the dominant microbiota population in the ND, HFD control, and PTE treatment groups at the phylum level. As expected, the alteration of gut microbiota composition by HFD feeding is significant (p < 0.0001). When mice fed a HFD were administered PTE for 5 weeks, the gut microbiota population was also significantly different from that of the HFD control (p < 0.05). Comparison of microbiota profiles in the ND and HFD control groups revealed that the Firmicutes population was increased in the HFD group, whereas the Bacteroidetes population was decreased, resulting in a reduction of the B/F ratio from 235.93% in the ND group to 34.62% in the HFD control group (Figure 6(c)). This result is consistent with several previous findings that Firmicutes are dominant in obese individuals [37,45]. Additionally, the lower B/F ratio in the HFD-fed mice was found to be mitigated to 62.73% by 5 weeks of PTE treatment. Moreover, the Proteobacteria population, which accounted for only 0.1% of the microbial population in the HFD control group, was 2% in the PTE treatment group. However, another dominant population, Deferribacteres, was 1.1% in the HFD control group, which was decreased to 0.2% by PTE treatment, a level similar to that of the ND group.

At the class level (Figure 6(d)), the differences in gut microbiota distribution between the ND group and HFD control and between the HFD control and PTE treatment group were both highly significant (p < 0.001). The most abundant bacterial population in the mouse gut at the class level was Bacteroidia. In this study, the Bacteroidia population accounted for 69.4% in the ND group, 25% in the HFD control group, and 37.1% in the PTE group. The Deferribacteres family, only 0.1% population in the ND group, was enriched in the HFD control group (1.1%) but decreased to 0.2% in the PTE group. Similarly, the Clostridia population was higher in the HFD control group (72.3%) but lower (58.9%) in the PTE group. Deltaproteobacteria, a reportedly abundant population in the gut, was present in relatively low levels in the HFD control group (0.1%), but substantially higher at 1.6% in the PTE treatment group. Treatment with PTE also increased the populations of Bacteroidaceae, Rikenellaceae, S24-7, Desulfovibrionaceae, and Enterobacteriaceae and decreased those of Lachnospiraceae, Ruminococcaceae, and Peptococcaceae (Figure 6(e)) compared with the HFD group. These results suggest that HFD feeding could dysregulate gut microbiota distribution in mice and that treatment with PTE could partially restore the microbiota distribution to one more similar to that of the ND group.

## Discussion

PTE is a commonly used herbal medicine in Chinese society. Treating fully differentiated 3T3L1 adipocytes with PTE decreased lipid accumulation in the cells by reducing lipid formation and TG content and enhancing lipase activity, which is encouraging. The inhibitory effect of PTE on lipid accumulation was also observed in HFD-induced obese mice and resulted in a smaller body size, reduced TG levels, lower body weight, and less epididymal fat after treatment with 250 mg/kg PTE for 5 weeks. The weight-gain inhibition rate, which is defined as [1 – (body weight gained in PTE treated mice)/(body weight gained in HFD control mice) × 100%], was 50.06% after 5 weeks of PTE treatment. This finding indicates that PTE is relatively potent than the clinically used drug orlistat (30.88%) in weight controling. [11]. Although PTE seems to be less effective than metformin (69.03%) [46], another clinically used anti-obesity drug, the duration of metformin treatment was 12 weeks, much longer than that of the PTE treatment (5 weeks) in this study. In addition, histological examination demonstrated that fat accumulation in the liver was attenuated by PTE treatment. All these findings strongly suggest that PTE contains anti-adipogenic compounds that warrant further investigation. Isolated phenylpropanoid-type metabolite tenuifoliside A [21] and triterpenoid saponin [22] from PTE were previously demonstrated to possess anti-inflammatory and antidepressant activities, respectively. Whether these compounds inhibit lipid accumulation remains unclear. Therefore, identification and purification of the active ingredients in PTE that inhibit lipid accumulation must still be performed.

Low toxicity is one of the key factors determining whether a compound can be developed into a drug. Even when 3T3-L1 cells were treated with a high concentration (500 μg/mL) of PTE, they did not exhibit obvious cytotoxicity. The levels of serum inflammatory markers, such as ALT, AST, and CR, were not elevated after mice were treated with PTE for 5 weeks. Furthermore, mice administered PTE remained active and had a similar feed intake as the control group. Together, these findings suggest that PTE is safe and could be a possible candidate with which to treat obesity. However, additional toxicology studies and clinical trials are required before PTE can be developed into a food supplement to moderate lipid accumulation.

The verification of genes differentially expressed in PTE-treated mice will help to explain how the plant extract inhibits lipid accumulation. Notably, one important transcription factor, PPARα, was activated after PTE treatment. PPARα is a ligand-activated transcription factor involved in fatty acid metabolism [47]. Earlier evidence indicated that PPARα^−/−^ mice had severe hepatic steatosis [48]. In particular, several genes upregulated by PTE, such as Lpin-1, Pctp, and Abcg5, were direct target genes of PPARα [49]. Lpin-1 encodes a phosphatidate phosphatase that prevents the accumulation of lipid intermediates in cells [50]. Pctp is a highly specific phospholipid lipid transfer protein. Studies concerning Pctp^−/−^ mice have found that body fat composition was increased in Pctp^−/−^ mice [49,51]. The function of Abcg5 is to maintain sterol balance in vertebrates [52]. More studies are required to clarify the compounds in PTE that may activate Lpin-1, Pctp, and Abcg5 gene expression through directly interaction with PPARα.

Several genes associated with obesity and anti-obesity treatment are downregulated by PTE, as has been reported in previous research. Mvd, a lipogenic enzyme, was downregulated in ob/ob mice receiving fatostatin treatment [53]. The expression of Pcsk9, associated with hypercholesterolemia, decreased after berberine treatment of HFD-induced obese mice [54]. The gene Serpina12 was found to be downregulated by *Peucedanum japonicum* extract in HFD-induced obese mice [55]. These results suggest that PTE inhibits lipid accumulation not only by lipid transfer and oxidation mediated by PPARα regulation, but also by inhibition of genes participating in lipogenesis and cholesterol biosynthesis. Although we have discovered differential gene expression in the livers of HFD-induced obese mice after PTE treatment, the effects of PTE on adipose tissue remain unclear. The complete mechanism of lipid accumulation inhibition of PTE will need to be further investigated.

Obesity has been recognized as an outcome of chronic low-grade inflammation. In the liver of HFD-induced obese mice, lipid accumulation activates Kupffer cells to release a large amounts of proinflammatory cytokines, such as TNFα, IL-1β, and IL-6 [56,57]. The effects of cytokines could be attenuated with clodronate to deplete Kupffer cells, resulting in a reduction of IL-1β, TG, and lipogenic enzyme expression levels. Conversely, treating primary mouse hepatocytes with IL-1β substantially increased TG content and fatty acid synthase expression [58]. Our study demonstrated that IL-1β expression was significantly inhibited by PTE treatment, which echoes a paper reporting that PTE decreased the production of TNFα and IL-1β [21]. Therefore, we hypothesize that PTE acts through a mechanism similar to anti-proinflammatory cytokine therapy, which was successful in improving glucose tolerance and insulin resistance in obese mice [59].

Strong evidence has been provided recently indicating that gut microbiota is an important factor affecting the development of obesity [60,61]. Gut microbiota distribution varied after prolonged HFD feeding in rats [32]. Changes in gut microbiota profiles may increase LPS release and thus cause chronic inflammation in obese patients [62]. These findings support the mechanism that obesity development is highly correlated with low-grade chronic inflammation and gut microbiota alteration. A HFD was shown to increase the Firmicutes population, which strengthens lipid droplet formation and absorption [63]. Additionally, a HFD causes a decrease in the Bacteroidetes population. This study elucidated that the reduced B/F ratio caused by a HFD could be partly restored by PTE treatment. In addition, 5-week PTE treatment decreased the population of Deferribacteres and increased that of Proteobacteria. These changes in gut microbiota profiles were similar to the evidence presented in previous reports for treating obesity with prebiotics (oligofructose) and antibiotics (vancomycin or penicillin) [64,65]. Furthermore, PTE treatment caused a reduction in both Lachnospiraceae and Ruminococcaceae populations, which positively correlate with obesity, in subjects fed a HFD [66,67]. In addition, PTE treatment restored the HFD-mediated reduction in the S24-7 population (a Bacteroidetes phylum), an effect also observed when subjects’ physical activity levels are increased [68].

## Conclusion

This study reports that PTE is an inhibitor of lipid accumulation in HFD-induced obese mice. After 5 weeks of treatment with PTE, increased body weight, elevated serum TG content, and liver steatosis were all reduced. Transcriptomic analysis revealed that genes involved in lipid and cholesterol biosynthesis and metabolism were altered after PTE treatment. The expression of the master transcription factor that regulates lipid oxidation (PPARα) was significantly higher relative to the HFD control mice. Furthermore, the low-grade chronic inflammation of the obesity caused by the HFD was also attenuated after PTE treatment, as demonstrated by the reduction of IL-1β expression level. In addition, treatment with PTE also restored the reduced B/F ratio caused by the HFD, enriched the Proteobacteria population, and reduced the Deferribacteres population. These shifts in particular gut microbiota populations are similar to those resulting from treatment of obese mice with prebiotics or antibiotics. These results suggest that PTE could be developed into a supplement to reduce lipid accumulation through attenuation of low-grade chronic inflammation and alteration of gut microbiota in obesity.

## Supplementary Material

ZFNR_A_1379861_Supp.zip

## Availability of data and materials

All data generated or analyzed during this study are included in this published article and its supplemental information files.

## Supplemental material

The supplemental material for this article can be accessed here.
