# Supplementary material for: Polygala tenuifolia extract inhibits lipid accumulation in 3T3-L1 adipocytes and high-fat diet–induced obese mouse model and affects hepatic transcriptome and gut microbiota profiles
Source: Food Nutr Res. 2017 Oct 5;61(1):1379861. doi: 10.1080/16546628.2017.1379861 (PMC5642193; doi:10.1080/16546628.2017.1379861)
Supplement: ZFNR_A_1379861_Supp.zip [file ZFNR_A_1379861_SM3293.zip › ZFNR_A_1379861_Supp/08. Additional Figure. S1_Revision_Final.docx]

**Fig. S1A**


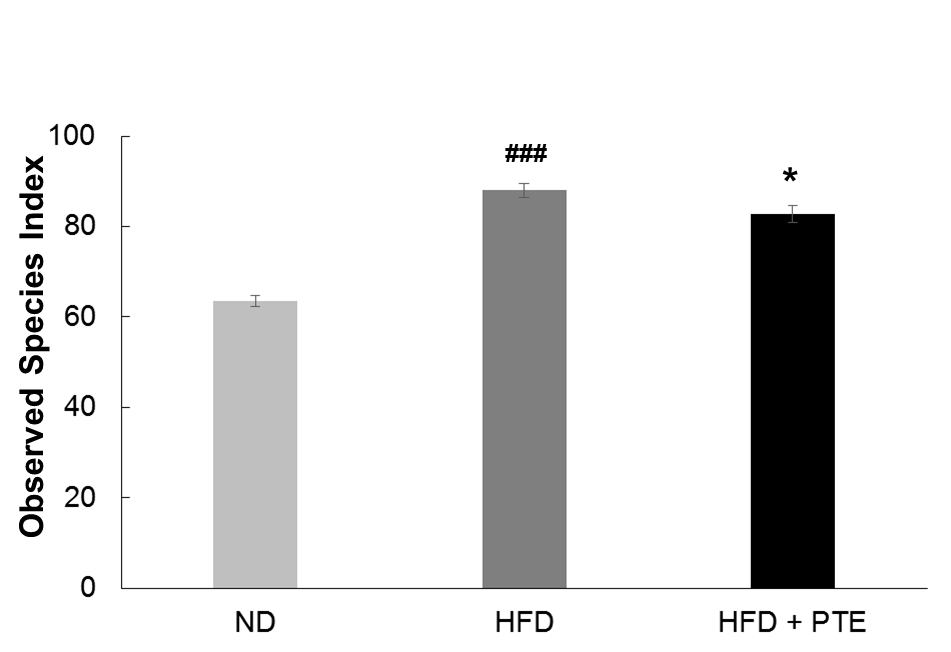


**Fig. S1B**


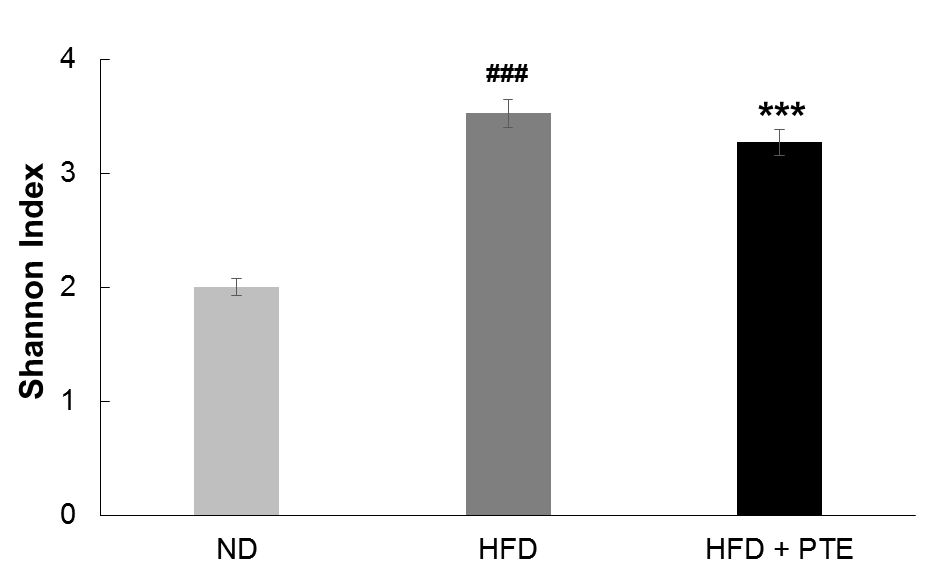


**Fig. S1. The diversity of the gut microbiota in ND group, HFD control and PTE treatments group.**

The richness index (number of observed species, Fig.S1A) and alpha diversity metrics (Shannon index, Fig.S1B) of each group were also calculated and followed by Pearson's chi-squared test. Differences were considered significant at p < 0.05 (^#^), p < 0.01 (^##^) and p < 0.001 (^###^) between ND group and HFD control group; p < 0.05 (*), p < 0.01 (**) and p < 0.001 (***) between PTE treated group and HFD control group.
