# Supplementary material for: Polygala tenuifolia extract inhibits lipid accumulation in 3T3-L1 adipocytes and high-fat diet–induced obese mouse model and affects hepatic transcriptome and gut microbiota profiles
Source: Food Nutr Res. 2017 Oct 5;61(1):1379861. doi: 10.1080/16546628.2017.1379861 (PMC5642193; doi:10.1080/16546628.2017.1379861)
Supplement: ZFNR_A_1379861_Supp.zip [file ZFNR_A_1379861_SM3293.zip › ZFNR_A_1379861_Supp/Additional Tables (S1A & S1B).docx]

**Additional files**

**Table S1A. Relative read numbers of 55 up-regulated genes**.

| **Gene** | **Fold Change*** | **C1**** | **C2***** | **Gene** | **Fold Change*** | **C1**** | **C2***** |
| --- | --- | --- | --- | --- | --- | --- | --- |
| Cd34 | 4.88 | 83.42 | 16.69 | H2-T9 | 2.09 | 300.93 | 143.93 |
| Pctp | 4.77 | 81.50 | 16.69 | Arl4d | 2.02 | 282.86 | 139.75 |
| Cela1 | 4.44 | 3083.68 | 694.58 | Klf12 | 2.00 | 371.08 | 185.64 |
| Cyp2a5 | 4.16 | 3361.65 | 807.16 | Mup12 | 1.96 | 4416.05 | 2252.25 |
| Hspa1a | 3.57 | 280.94 | 78.37 | Fabp5 | 1.94 | 353.82 | 182.51 |
| Tff3 | 3.43 | 97.80 | 28.16 | Pfkfb3 | 1.92 | 389.30 | 202.32 |
| Rassf4 | 3.42 | 365.32 | 106.38 | Pcp4l1 | 1.90 | 303.96 | 159.57 |
| Lpin1 | 3.29 | 3409.69 | 1036.65 | Arid5b | 1.88 | 419.98 | 223.18 |
| Fam171b | 3.26 | 194.65 | 59.45 | 1810011O10Rik | 1.84 | 291.49 | 158.52 |
| Afap1l1 | 3.14 | 95.89 | 30.24 | Eif4ebp3 | 1.80 | 769.96 | 426.55 |
| Rgs16 | 2.99 | 506.28 | 168.95 | Mbd1 | 1.80 | 596.41 | 331.65 |
| Cyp2a22 | 2.92 | 59.86 | 20.17 | Hsd17b6 | 1.79 | 3784.75 | 2117.09 |
| Vsig10 | 2.85 | 63.28 | 21.90 | Onecut1 | 1.75 | 252.18 | 143.92 |
| Cyp39a1 | 2.83 | 77.67 | 27.12 | Igfbp2 | 1.73 | 2948.48 | 1703.07 |
| Hspa1b | 2.82 | 473.53 | 167.72 | G6pc | 1.73 | 2204.41 | 1275.48 |
| Cfap53 | 2.76 | 75.75 | 27.12 | Nr0b2 | 1.71 | 485.18 | 283.67 |
| Grem2 | 2.76 | 374.91 | 135.58 | Camk1d | 1.65 | 481.35 | 290.97 |
| Akr1e1 | 2.64 | 464.09 | 175.21 | Fkbp5 | 1.64 | 871.60 | 529.80 |
| Ahcy | 2.43 | 5533.12 | 2272.21 | Gpcpd1 | 1.63 | 1680.87 | 1031.44 |
| Lyve1 | 2.36 | 251.22 | 106.38 | Fgfr2 | 1.63 | 440.11 | 270.11 |
| Alas1 | 2.33 | 4251.56 | 1826.14 | Slc38a2 | 1.62 | 1379.79 | 851.01 |
| Ppara | 2.28 | 31.64 | 15.56 | Abcg5 | 1.62 | 1086.38 | 670.59 |
| Cyp2a12 | 2.26 | 4994.26 | 2207.49 | Rnf169 | 1.61 | 494.77 | 306.62 |
| Plxna2 | 2.23 | 377.79 | 168.95 | Cyp3a11 | 1.61 | 18335.2 | 11418.8 |
| Rsph3b | 2.20 | 127.46 | 57.70 | Upp2 | 1.55 | 5129.87 | 3303.94 |
| Ceacam2 | 2.14 | 241.96 | 112.59 |  |  |  |  |
| Smad9 | 2.13 | 176.43 | 82.39 |  |  |  |  |
| Gm10052 | 2.12 | 225.25 | 106.18 |  |  |  |  |
| Dsg1c | 2.10 | 227.21 | 107.93 |  |  |  |  |
| Slco1a4 | 2.10 | 158.35 | 75.19 |  |  |  |  |

* Fold change, the ratio of PTE group to HFD group.

**C1 mean, reads number of the genes in PTE treatment group.

***C2 mean, reads number of the genes in HFD control group.

**Table S1B. Relative read numbers of 72 down-regulated genes**.

| **Gene** | **Fold Change*** |  | **C1**** | **C2***** | **Gene** | **Fold Change*** | **C1**** | **C2***** |
| --- | --- | --- | --- | --- | --- | --- | --- | --- |
| Rpl14-ps1 | 0.05 |  | 20.45 | 431.94 | Hist1h2al | 0.46 | 79.79 | 172.39 |
| Marco | 0.07 |  | 52.74 | 722.74 | Dynlt1b | 0.47 | 55.43 | 119.42 |
| BC021614 | 0.12 |  | 125.61 | 1081.50 | Saa1 | 0.48 | 3685.80 | 7728.05 |
| Creld2 | 0.23 |  | 267.52 | 1160.76 | Idi1 | 0.49 | 693.25 | 1427.74 |
| Mup19 | 0.24 |  | 4828.60 | 20095.74 | Hba-a1 | 0.49 | 2152.56 | 4407.23 |
| 2310057J18Rik | 0.26 |  | 35.48 | 137.66 | Pcsk9 | 0.50 | 131.36 | 262.81 |
| Myom3 | 0.26 |  | 20.14 | 78.22 | Gm14296 | 0.51 | 151.14 | 296.36 |
| Mvd | 0.28 |  | 31.64 | 113.68 | Magt1 | 0.51 | 936.76 | 1821.20 |
| Il1b | 0.30 |  | 17.26 | 59.45 | Neat1 | 0.52 | 607.91 | 1175.36 |
| Sqle | 0.31 |  | 109.31 | 357.72 | Eno1b | 0.52 | 468.67 | 897.43 |
| Pira1 | 0.31 |  | 34.75 | 111.69 | Nt5e | 0.52 | 100.68 | 192.94 |
| Fam25c | 0.31 |  | 48.90 | 156.44 | Cyp51 | 0.53 | 334.64 | 633.05 |
| Lcn2 | 0.32 |  | 148.62 | 460.97 | Glo1 | 0.54 | 4524.84 | 8446.53 |
| Cyp2d12 | 0.35 |  | 32.11 | 93.91 | Hbb-b2 | 0.54 | 239.41 | 442.99 |
| Slc17a9 | 0.35 |  | 31.64 | 90.73 | Hbb-bt | 0.54 | 239.41 | 442.99 |
| Gale | 0.36 |  | 52.74 | 148.09 | Gbp11 | 0.54 | 995.42 | 1833.21 |
| Igsf23 | 0.37 |  | 28.77 | 78.22 | Ccbl2 | 0.54 | 830.37 | 1528.91 |
| Serpina3g | 0.38 |  | 59.60 | 156.74 | Apol9a | 0.54 | 368.67 | 677.67 |
| Xlr3a | 0.39 |  | 43.73 | 111.79 | Hp | 0.55 | 28910.44 | 52241.47 |
| Rsc1a1 | 0.40 |  | 73.33 | 185.99 | Hbb-b1 | 0.55 | 3015.25 | 5439.29 |
| Igsf6 | 0.40 |  | 36.44 | 90.73 | Slc15a4 | 0.56 | 344.23 | 619.49 |
| Acnat2 | 0.41 |  | 145.28 | 358.63 | Mup18 | 0.56 | 1550.45 | 2786.06 |
| Syvn1 | 0.41 |  | 497.65 | 1214.99 | Pdia4 | 0.57 | 1394.18 | 2446.67 |
| Rhbg | 0.42 |  | 77.67 | 187.72 | Cdk2ap2 | 0.57 | 313.55 | 547.53 |
| Eif3j2 | 0.42 |  | 281.67 | 673.51 | Mug2 | 0.58 | 2614.16 | 4513.35 |
| Saa2 | 0.42 |  | 1568.73 | 3739.78 | Hsp90b1 | 0.59 | 6533.64 | 11048.59 |
| Lad1 | 0.42 |  | 37.40 | 89.69 | Hmox1 | 0.60 | 295.33 | 495.38 |
| Hyou1 | 0.42 |  | 1113.23 | 2648.99 | Leap2 | 0.61 | 457.37 | 756.11 |
| Pqlc1 | 0.43 |  | 324.09 | 758.20 | Mlec | 0.61 | 1121.86 | 1828.22 |
| Manf | 0.43 |  | 695.17 | 1601.91 | Oat | 0.61 | 1425.82 | 2320.48 |
| Fdps | 0.44 |  | 519.70 | 1183.70 | Serpina12 | 0.62 | 1674.16 | 2704.27 |
| Derl3 | 0.44 |  | 89.17 | 201.28 | Lars2 | 0.63 | 14175.71 | 22620.73 |
| Tubb2a | 0.45 |  | 166.51 | 368.89 | Msmo1 | 0.63 | 756.54 | 1203.52 |
| Dct | 0.46 |  | 95.89 | 209.63 | Elovl3 | 0.63 | 715.31 | 1129.47 |
| Sdf2l1 | 0.46 |  | 352.86 | 766.54 | Lbp | 0.64 | 966.53 | 1512.22 |
| Hspa5 | 0.46 |  | 6172.15 | 13378.46 | Cd5l | 0.65 | 1542.80 | 2373.66 |

* Fold change, the ratio of PTE group to HFD group.

**C1 mean, reads number of the genes in PTE treatment group.

***C2 mean, reads number of the genes in HFD control group.
